# Amino acids biosynthesis in root hair development: a mini-review

**DOI:** 10.1042/BST20231558

**Published:** 2024-07-10

**Authors:** Jesús Montiel, Joseph G. Dubrovsky

**Affiliations:** 1 Departamento de Genómica Funcional de Eucariotas, Centro de Ciencias Genómicas, Universidad Nacional Autónoma de México, Cuernavaca 62210, Mexico; 2 Departamento de Biología Molecular de Plantas, Instituto de Biotecnología, Universidad Nacional Autónoma de México, Cuernavaca 62210, Mexico

**Keywords:** amino acids, *Arabidopsis thaliana*, epidermis, metabolism, root development, root hairs

## Abstract

Metabolic factors are essential for developmental biology of an organism. In plants, roots fulfill important functions, in part due to the development of specific epidermal cells, called hair cells that form root hairs (RHs) responsible for water and mineral uptake. RH development consists in (a) patterning processes involved in formation of hair and non-hair cells developed from trichoblasts and atrichoblasts; (b) RH initiation; and (c) apical (tip) growth of the RH. Here we review how these processes depend on pools of different amino acids and what is known about RH phenotypes of mutants disrupted in amino acid biosynthesis. This analysis shows that some amino acids, particularly aromatic ones, are required for RH apical (tip) growth, and that not much is known about the role of amino acids at earlier stages of RH formation. We also address the role of amino acids in rhizosphere, inhibitory and stimulating effects of amino acids on RH growth, amino acids as N source in plant nutrition, and amino acid transporters and their expression in the RHs. Amino acids form conjugates with auxin, a hormone essential for RH growth, and respective genes are overviewed. Finally, we outline missing links and envision some perspectives in the field.

## Introduction

The sessile nature of plant organisms is directly related to their specific developmental and physiological adaptations to the environment. The autotrophic aerial parts of plants provide energy to heterotrophic underground organs, including roots. The latter explore soil or substrate where a plant grows and provides the plant organism with water and minerals. Epidermis is a tissue at the interface between plant organism and the environment, and in the root it performs many functions, including its participation in uptake of salts and water, interaction with rhizosphere organisms, function as barrier that facilitates avoidance of toxic compounds and uptake of xenobiotics; it also facilitates anchoring function of the root [1–5]. All these functions are accomplished with the aid of root hairs (RHs). At the whole plan level, the RHs highly increase the root surface and so their development is essential for proper root functions. For example, calculated total surface of only RHs (excluding non-RH surface) of a single rye plant roots is equal to 401 m^2^ [6].

The root epidermal cells are produced in the root apical meristem, whose cells are continuously displaced to the elongation zone where in majority of species cells elongate more than 20-fold [7]. When their growth is almost completed, epidermal cells near rootward transverse cell wall start to form thin tubular extensions that are a few times longer than the cells from which they are formed and can reach 1.5 mm in length [5]. This epidermal cell extension is called RH, and the respective epidermal cells are called root-hair cells as opposed to epidermal cells that do not develop RHs (non-hair cells). The epidermal cells in the root apical meristem that are committed to form RHs are called trichoblasts and cells which will not form RHs are atrichoblasts. Sometimes, the term trichoblast is incorrectly used for RHs themselves [8], or RH-bearing cells [9], and to avoid this confusion it is essential to consider that trichoblast and atrichoblast cells are in the meristematic state and hair and non-hair cells are differentiated cells formed from these cells that only after their complete elongation start (or do not) RH formation. The determination of developmental fate takes place during the last cell cycle in the root apical meristem and soon after [10–13]. In some species, all epidermal cells form RHs, but in most cases hair cells are interspersed with non-hair cells in different patterns, depending on taxon [3,14–16]. So, the RH development is predetermined in the root apical meristem. Then the RH formation starts from the initiation phase when a RH bulge is formed by hair cell. This phase is followed by rapid elongation of the RH maintained due to apical (or tip) growth taking place near the apex of the hair extension [13,17]. Gene regulatory networks for trichoblast and atrichoblast determination are complex and depend on interplay between transcription factors, leucine-rich repeat receptor-like kinases, other factors, hormones, and environmental factors, such a mineral deficiency (reviewed in [3,13,18–20]). Among multiple factors involved in the RH formation, perhaps the least attention was paid to the role of amino acids.

## Biological processes affected in mutants disrupted in amino acid biosynthesis

**Figure 1.**
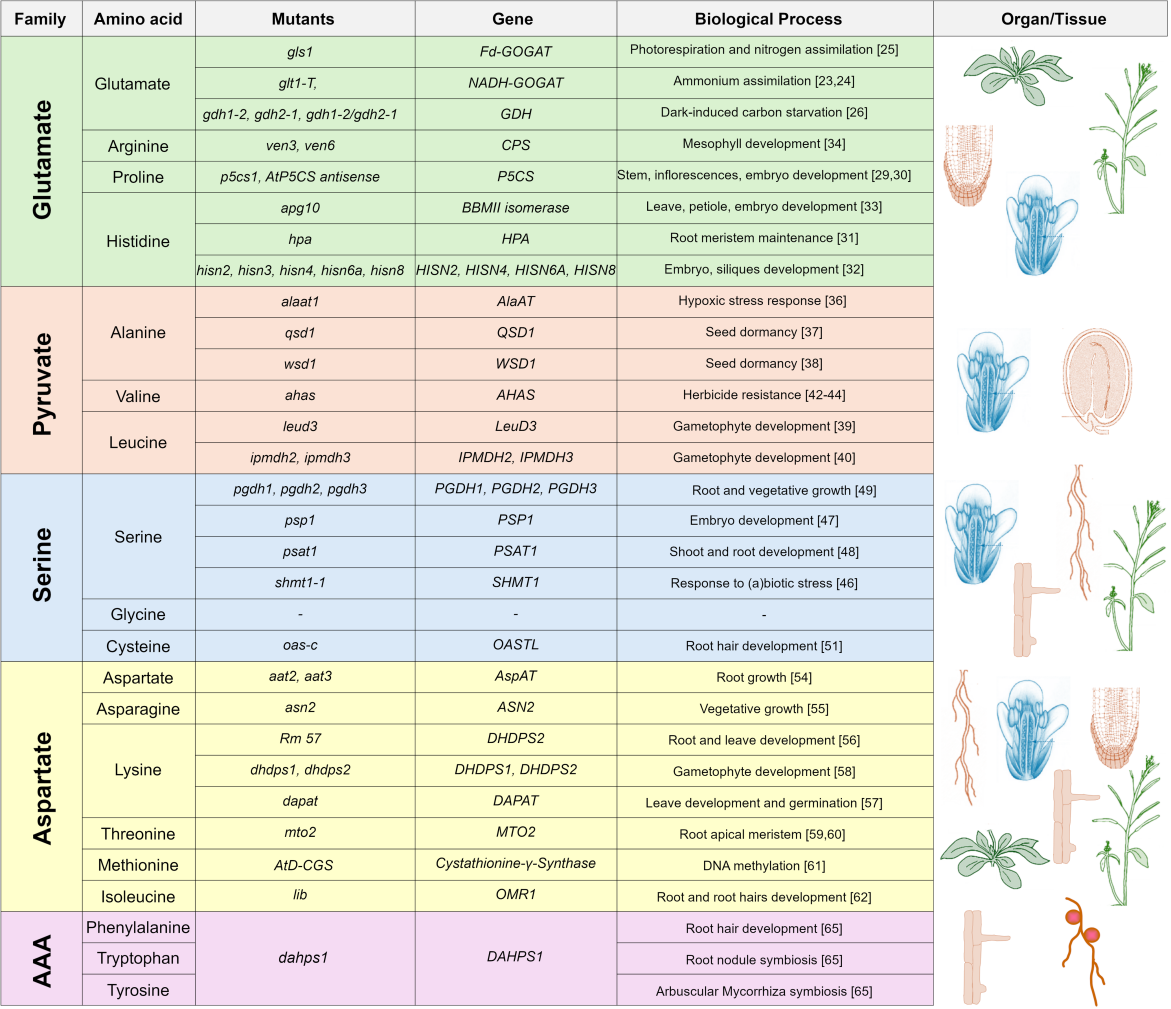
Biological processes linked to amino acid biosynthesis. Five families of amino acid biosynthesis pathways and respective mutants affected in genes encoding enzymes participating in these pathways: Glutamate (green), Pyruvate (Pink), Serine (blue), Aspartate (yellow) and Aromatic Amino Acids (AAA, purple). The organ or tissue where respective pathways are most important and related biological processes and developmental programs are outlined.

Amino acid biosynthesis comprises a complex metabolic network, occurring in different subcellular compartments [21]. In the last few decades, numerous studies have addressed the role of different amino acids in various biological processes by analyzing the phenotype of mutants disrupted in different steps of amino acid biosynthesis pathways. Depending on the precursors, amino acids can be categorized into 5 families, derived from glutamate, pyruvate, serine, aspartate, or chorismate [21] (Figure 1), and here we briefly outline their role in developmental processes, including RH formation. When not specified, we always refer to L-isoform of amino acids.

### Glutamate family

Glutamate is a primary amino acid that serves as an N-donor for proline, glutamine, arginine, and histidine biosynthesis. In plastids, Glu is mainly produced by the activity of glutamate synthase (known as GOGAT), and glutamate dehydrogenase [22]. Several studies reveal that mutation of genes encoding these enzymes provoke an imbalance in nitrogen and carbon metabolism [23–26]. Glu was shown to cause root growth inhibition via shortening of the root apical meristem and the elongations zone resulted in RH development very close to the root apex [27]. On the other hand, mutants disrupted in Arg, Pro, and His biosynthesis display developmental abnormalities both in underground and aerial organs [28–35].

### Pyruvate family

Pyruvate can be converted into the proteinogenic amino acid alanine by alanine aminotransferases (*AlaAT*). In *Arabidopsis thaliana*, the hypoxic stress response is compromised in the *alaat1*-1 mutant [36]. Moreover, in rice and barley, seed dormancy is perturbed in mutants affected in *AlaAT* genes [37,38]. Similarly in *A. thaliana*, defects in male and female gametophyte development are observed in mutant lines interfered in different steps of leucine biosynthesis, a pyruvate-derived amino acid [39,40]. Valine is a branched-chain amino acid, which is synthesized from pyruvate with a set of four enzymes, where acetohydroxyacid synthase (*AHAS*) is the first in the biosynthetic pathway [41]. In several plant species, mutations in *AHAS* isoforms confer resistance to herbicides [42–44].

### Serine family

The phosphorylated glycerate and glycolate pathways lead to serine formation, and this amino acid can be converted into glycine and cysteine [45]. Root growth, embryo development and responses to (a)biotic stress are compromised in mutants disrupted in Ser biosynthesis [46–49]. However, there is evidence that interfering with Cys anabolism has a negative impact on RH growth. In the cytosol, plastid, or mitochondrion, Cys is synthesized by a hetero-oligomeric cysteine synthase complex, composed by serine acetyltransferase, and O-acetylserine(thiol)lyase (OASTL) [50]. In *A. thaliana*, RH length is severely reduced in *oas-c* mutants [51]. This phenotype is apparently linked to the key role of Cys in the detoxification process of the phytotoxic molecule cyanide, a byproduct generated during biosynthesis of a plant hormone ethylene. In non-cyanogenic species, β-cyanoalanine synthase (β-CAS) catalyzes the conversion of Cys and cyanide into β-CAS, and hydrogen sulfide [50]. In the β-CAS mutant, *cys-c1*, cyanide is abundantly accumulated in the roots, where RHs emerge normally but the elongation process is arrested. Similar defects in RH development are observed when wild type plants are incubated with KCN, reinforcing the relevance of cyanide detoxification during RH development [52].

### Aspartate family

Aspartate is the precursor of the Aspartate family amino acids, constituted by asparagine, aspartate, lysine, methionine, threonine, and isoleucine. Asp is synthesized from oxaloacetate by aspartate aminotransferases (AspAT) [53]. In *A. thaliana*, mutants affected in the cytosolic (AAT2) or chloroplastic (AAT3) AspAT, have altered Asp levels and reduced root growth [54]. On contrary, the phenotype reported for an asparagine synthetase mutant in *A. thaliana* (*Atasn2*-2), indicates that Asn plays an important role in vegetative growth [55]. Similarly, the root and overall plant growth are affected in mutants disrupted in key steps of Lys biosynthesis [56–58]. In a loss-of-function mutant affected in *THREONINE SYNTHASE1* gene, also called *METHIONINE OVERACCUMULATOR2*, the root apical meristem activity in *Arabidopsis* roots becomes completely compromised and all meristematic cells differentiate resulting in RH formation at the very tip [59,60], which are also shorter than in wild type (unpublished results). On the other hand, an imbalance of Met metabolism, expressing a feedback-insensitive form of cystathionine γ-synthase (*AtD-CGS*), the key gene of Met synthesis, leads to high Met content that impacts the DNA methylation profile of DNA in *A. thaliana* [61]. Root development is severely compromised in the *lib* mutant (low isoleucine biosynthesis), disrupted in the *OMR1* gene that encodes a threonine deaminase/dehydratase (TD), which catalyzes the first and also the committed step towards isoleucine biosynthesis. Additionally, the emergence of RHs in the *lib* mutant was unequally distributed, with some root portions lacking RHs. In the maturation zone the scarce RHs in the *lib* mutant were ∼70% shorter than those in wildtype [62].

## Aromatic amino acids

Phenylalanine, tyrosine, and tryptophan constitute the group of aromatic amino acids (AAA), which are synthesized via the shikimate pathway, a seven-step metabolic route. The first enzyme in this pathway is the plastidic 3-deoxy-d-arabino-heptulosonate-7-phosphate synthase (DAHPS), that catalyzes the formation of 3-dehydroquaianate from PEP and E4-P [63]. In *A. thaliana*, three DAHPS isoforms exist, and single mutants disrupted in each of the respective genes have root length comparable to that in wild-type plants [64]. Similarly, the model legume *Lotus japonicus* contains three *DAHPS* genes, and the root growth is not significantly affected in the *Ljdahps1* mutant. However, the RH development is dramatically altered in *Ljdahps1*. Although RHs emerge normally in the differentiation zone, the tubular morphology of these cells is perturbed soon after emergence, forming balloon-like structures that eventually collapse [65]. Additional evidence indicates that the disruption of the actin cytoskeleton dynamics and cell wall integrity found in the *Ljdahps1* mutant is likely related to the progressive loss of apical growth of the RHs. The interplay of AAA with the cell wall biomechanics of RHs is further supported by RNAseq data, since 19 genes related to cell wall functioning were significantly down-regulated in the *Ljdahps1* roots. It is worth noting that although *LjDAHPS1* expression was detected in different root tissues, only the RH development was clearly affected, compared with other root tissues, revealing a preponderant role of AAA biosynthesis during RH growth [65].

## Amino acids in the rhizosphere and RHs

During growth, plant roots exude different organic compounds, among which amino acids and amino acid analogues are very common [66–69]. Amino acids is a proven N source for plants growing *in situ* and they can be retained in soils at relatively high levels [70,71]. Interestingly, a RH-less maize mutant deficient in RH elongation exudes a greater amount of amino acids [69]. The authors propose that this increase compensates ‘the lower absorption surface by increasing nutrient mobility’ [69]. A toxic nonprotein amino acid, L-canavanine (l-2-amino-4-(guanidinooxy) butyric acid), is found in certain leguminous plants in high quantities and show antibacterial properties [72]. This amino acid is exuded by licorice (*Glycyrrhiza uralensis*) roots and its presence is essential for *Mesorhizobium tianshanense* survival in the RH microenvironment and for control of bacterial rhizosphere diversity to establish symbiosis between a legume and rhizobia [66].

## Amino acids as a nitrogen source that affects RH formation

Amino acids can promote RH growth. Exogenous treatment of tobacco plants with 0.1 µM Gly promotes RH tip growth, and their length is two-fold greater than in untreated plants; this increase is accompanied with an increased expression of genes involved in RH formation [73]. A wide screening of a variety of aminos acids (Ala, Arg, Asn, Asp, Cys, Glu, Gln, Gly, His, Ile, Lys, Met, Phe, Pro, Ser, Thr, Trp, Tyr, Val, Leu; both, L and D isomers) was performed on *Brassica rapa* plants addressing RH density [74]. In this study, the only amino acids that showed an increase in this parameter were L-Met and D-Met. Interestingly, both isomers induced a similar increase in RH density (that reached three-fold at 67.0 µM amino-acid concentration compared with untreated plants), but D-Met at a lower concentration (13.4 µM) was much more biologically active than L-Met [74]. What are the cellular bases of this effect and whether the density increase is accompanied with changes in RH growth is not known. In a different study it was shown that both RH density and length were increased more than two-fold when *B. rapa* grew in presence of degraded bluegill (fish from order *Parcifomes*) products that contain a mixture of peptides and amino acids [75]. This study shows that fish waist can be used for plant growth stimulation and that this growth promotion is mediated by RH development. Most probably the outlined effects of amino acid mixture are not specific but result from the uses of amino acids as additional nitrogen source.

Some amino acids can exert inhibitory effect on root growth. For example, when *A. thaliana* seedlings grew on medium supplemented with Asn as a sole N source, their root length was 61 and 30% (2.5 and 10 mM) of that in seedlings that grew without N source [76]. The RH development is also inhibited by Asn. In the same study it was shown that Asn catabolism is involved in a decrease of this inhibitory effect. Asparaginase genes *ASPGA1* and *ASPGB1* encode enzymes that degrade Asn to aspartic acid and ammonia. The RH formation in a double mutant in these genes is inhibited to a much greater extent in presence of exogenous Asn (3.5 and 5 mM); at these concentrations, by three days of growth in wild type, 97% and 46% of seedling were with the RHs, while only 25 and 8% of *aspga1-1 aspgb1-1* double mutant seedlings formed the RHs [76].

The RHs are amenable to single cell type analysis as only RH fraction of hair-cells can be collected [77,78]. This approach permits proteomic and transcriptomic analyses and quantification of relative protein expression under optimal nutrient conditions and when a single nutrient is missing. Such an approach in young maize (*Zea mays*) seedlings showed that in N-deprived medium, amino acid (Met and aromatics) synthesis is up-regulated, whereas their degradation is down-regulated [79].

## Amino acid transport

To be transported insight the root, amino acids should pass from the rhizosphere through the epidermis simplasmically [80,81]. The RHs increase root surface and thereby facilitate this transport. In *A. thaliana*, a minimum of 53 amino acid transporters are identified [82]. As expected, amino acid transporters are predominantly expressed in epidermis, including the RHs. For example, *AMINO ACID PERMEASE 1* (*AAP1*, At1g58360) promoter is active in epidermis, including the RHs; it is also active in the ground tissue and central cylinder of both, the root apical meristem and the elongation zone [83]. The protein is localized at the plasma membrane predominantly in the epidermis. Interestingly, related *aap1* mutants have a reduced capacity for amino acid uptake and are capable to grow in media supplemented with high (toxic) concentrations of several amino acids [83]. The *AMINO ACID PERMEASE 5* (*AAP5*, AT1G44100) was shown to be responsible for basic amino acid (specifically, Arg and Lys) transport into the root tissues [84]. These studies show that AAP1 and AAP5 are essential for organic nitrogen acquisition, specifically, for uptake by roots of neutral amino acids, Glu, and His and they are capable to transport amino acids at relatively low concentrations present in soil [84,85]. Another amino acid transporter, LYSINE HISTIDINE TRANSPORTER1, (LHT1, AT5G40780) is involved in Gln, Ala, Glu and Asp uptake in *A. thaliana* roots, especially when present at low soil concentrations, which can be as low as 2 µM [84]. Importantly, this transporter is also predominantly expressed in epidermis and its expression increases sharply starting from the young differentiation zone where the RHs are formed [86,87]. A related *LHT6* (At3g01760) *A. thaliana* gene, expressed in root tissues, including the RHs, encodes a transporter involved in uptake of acidic amino acids, Ala and Glu [85]. Rice (*Oryza sativa* L.) roots also show the highest expression level of *OsLHT1* in the RHs; in this species this transporter is involved in the transport of Asp, Asn and Glu [88].

## Other links between RHs and amino acids

Some amino acids were shown to exert stress-resistant role. A lower fraction of the RHs in *A. thaliana* seedlings subjected to heat stress was undergoing necrosis when treated with 1 and 5 µM Pro, compared with untreated control [89]. The authors propose that this protective Pro effect can be related to its antioxidant glutathione-mediated action involved in maintenance of redox balance [89].

**Figure 2.**
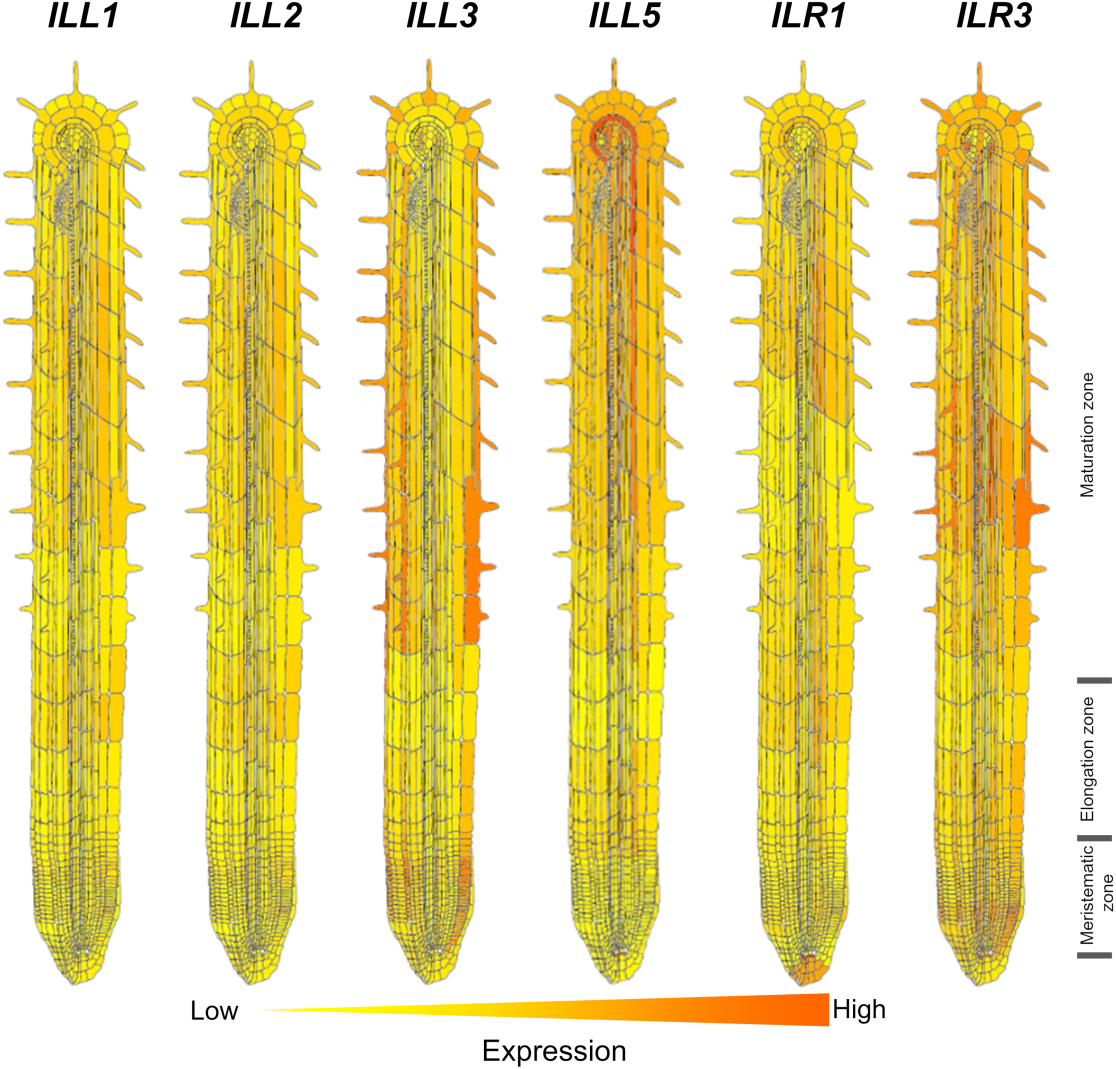
Expression pattern of some of the IAA-amino acid hydrolases in *Arabidopsis thaliana* in accordance with references [81,82]. The gene names are as follows: IAA-LEUCINE RESISTANT (ILR)-LIKE GENE (*ILL1,* AT5G56650), *ILL2* (AT5G56660), *ILL3* (AT5G54140), *ILL5* (AT1G51780), IAA-LEUCINE RESISTANT1 (*ILR1*, AT3G02875), *ILR3* (AT5G54680*)*. Note that practically all genes show longitudinal gradient with an increase from the root apical meristem (meristematic zone) towards the maturation (differentiation) zone of the root. Gene expression is notably higher in hair cells for *ILL1*, *ILL2*, *ILL3*, and *ILR3*.

The growth of RH depends on auxins [17,90–92]. Auxin metabolism and homeostasis involves auxin storage forms that are related to the RH growth [93,94]. One of the auxin (its most common form is indole-3-acetic acid, IAA) storage forms is present in form of conjugates with amino acids [95,96]. Amidohydrolases are required to liberate free auxin from its conjugated forms such as IAA-Leu, IAA-Phe, and IAA-Ala [95,97] to exert its action of the RH growth. *IAA–LEUCINE RESISTANT1* (*ILR1*, AT3G02875) encodes an amidohydrolase that cleaves IAA–Leu; *IAA–ALANINE RESISTANT3* (*IAR3,* AT1G51760) encodes a protein involved in hydrolysis of IAA-Ala; and the *ILR1-LIKE PROTEIN2* (*ILL2,* AT5G56660) also encodes an IAA-Ala hydrolase [93,95]. Seedlings of triple mutants affected in these genes show two-fold reduction in free auxin content and about two-fold increase in IAA-Ala content [96]. In agreement with these changes, the RH length in this mutant is 30% shorter than in wild type [93]. Interestingly, revision of expression pattern of *ILR1*, *IAR3*, and *ILL2* shows that, though they are not exclusively expressed in the epidermis, a clear increase in their expression is observed in the young differentiation zone where the RH are formed, while the expression level in all tissues within the root apical meristem and the elongation zone is low or undetectable [86,87]. Some other IAA-amino acid hydrolases are also present in *A. thaliana* genome. Though their role in the RH development was not studied, similarly to the reported genes, they show much greater level of expression in the epidermis of the young differentiation zone compared with growing part of the root (Figure 2; *ILL1*, *ILL3*, *ILL5*, *ILR3*, and *ILL2*). It is also peculiar that *ILL3* and *ILR3* show in addition to outlined gradient, a greater expression level in hair cells compared with non-hair cells [86,87]. These findings clearly suggest that fine-tuning of auxin level in epidermis can be decisive for RH growth.

Auxin catabolism of amino-acid-conjugated auxin can additionally control local auxin concentration and thereby co-ordinate RH growth. The DIOXYGENASE FOR AUXIN OXIDATION 1 (AtDAO1) enzyme promotes oxidation of auxin, apparently, in both, its free and conjugation form. Auxin becomes converted to 2-oxoindole-3-acetic acid (oxIAA). Loss-of-function mutants affected in *AtDAO1* show levels of auxin-Asp conjugates up to three orders of magnitude higher than those in wild type, but the auxin level is not changed. Importantly, the RH length increases by 80% in *dao1* mutants [98,99]. The exact mechanism of this RH growth promotion is not yet completely clear. No data are available on whether auxin conjugation or deconjugation is also involved in trichoblast and atrichoblast patterning in the root apical meristem. However, mathematical modeling suggests that in *dao1*-1 mutant, the level of free auxin is increasing compared with wild type specifically in the epidermis of the root apical meristem [100] suggesting that changed balance between IAA, oxIAA, and oxIAA-ASP may affect cell fate determination. New studies are needed to understand how conjugation of free auxin with amino acids and their catabolism are regulated and whether new amino acid synthesis is required for the conjugate formation. Another unexplored question is what the possible role of amino acids is when liberated from IAA-amino acid conjugates and whether they are also involved in modulation of RH formation.

## Perspectives

- Amino acids are indispensable molecules for living organisms, responsible for plethora of functions. In plants, amino acids are synthesized in several cellular compartments through complex interconnected metabolic routes, that have impeded a comprehensive analysis of their role in different biological processes or responses towards (a)biotic stimuli. Importantly, different lines of evidence indicate a preferential role for certain amino acids in specific developmental programs, such as RH growth.
- Here, we outlined the links between pools of certain amino acids and the RH formation, and now it is clear that metabolic availability of at least some free amino acids is decisive for the RH development. Despite that, it has to be recognized that amino acid action on the RH formation can be indirect and result from their effect on root growth processes, related to cell proliferation, hormonal signaling, and metabolic effects. Therefore, it is still unclear what is the contribution of some amino acids to RH development, but this incognita can be addressed by analyzing the RH formation in the wide collection of mutants interfered in the biosynthetic pathways for amino acid biosynthesis.
- Additionally, it becomes relevant to unveil the complex interplay of amino acids with other molecular components of the cell. Also, how specific processes of RH development, such as trichoblast/atrichoblast cell fate determination, RH initiation phase or apical (tip) growth of the hair itself depend on amino acids is yet to be established. More challenging questions also await clarifications. For example, single-cell metabolomics [101–103] could help to address the role of specific amino acids in specific aspects of the RH development. The study of dynamic aspects of amino acid metabolism is also demanding. We do not yet know how fast a newly synthesized amino acid molecules are incorporated in protein synthesis, how a pool of free amino acids in a cell is established and regulated, and what are the dynamic aspects of cell-to-cell or long-distance amino acid transport among different cell types and plant organs. Answers to all these questions will help us to comprehend RH formation among other aspects of root development.

## Abbreviations

AAA: aromatic amino acid
AHAS: acetohydroxyacid synthase
AlaAT: alanine aminotransferases
AspAT: aspartate aminotransferases
β-CAS: β-cyanoalanine synthase
DAHPS: 3-deoxy-d-arabino-heptulosonate-7-phosphate synthase
IAA: indole-3-acetic acid
RH: root hair
